# Case Report: Carcinoid heart disease with severe tricuspid regurgitation and concomitant patent foramen ovale causing severe hypoxia

**DOI:** 10.3389/fcvm.2023.1309929

**Published:** 2024-01-18

**Authors:** Michael Cronin, Brendan McAdam

**Affiliations:** Department of Cardiology, Beaumont Hospital, Dublin, Republic of Ireland

**Keywords:** carcinoid heart disease, tricuspid regurgitation, patent foramen ovale, transcatheter, echocardiography

## Abstract

This case report demonstrates a unique case of managing complex concomitant structural cardiac issues using transcatheter techniques in a frail patient. The primary regurgitant lesion in this case caused significant right to left shunting with severely debilitating hypoxaemia for the patient, requiring high volumes of ambulatory oxygen to compensate. We would like to highlight the role of multi-modality cardiac imaging demonstrated in this case, as well as the limited surgical data and poor outcomes in advanced disease with higher peri-operative complications. Finally, it should be noted that percutaneous correction of structural lesions may provide palliative relief but carries an uncertain risk of recurrence.

## Introduction

Carcinoid heart disease (CHD) is a recognised rare cause of right-sided valvular heart disease (VHD) and remains a major cause of morbidity and mortality in the cardio-oncology population. This case demonstrates CHD complicated by tricuspid regurgitation (TR) causing right to left heart shunting via a stretched patent foramen ovale (PFO), with marked hypoxia and severe heart failure. The tricuspid valve (TV) has a complex anatomy that is essential to understanding the pathophysiology of TR in CHD (1), and multi-modal imaging is required in its evaluation, including transthoracic echocardiography (TTE), transoesophageal echocardiography (TOE), cardiac computed tomography (CT), and invasive catheterisation with fluoroscopy, as demonstrated in this clinical case. There remains a limited specific advice regarding the management of VHD in CHD, with poor surgical outcomes, low procedural numbers, and high anaesthetic risk in this frail population (2, 3). Our case demonstrates the narrow clinical window regarding intervention, when the TR is severe but the right ventricular (RV) function is intact. Secondary to the associated frailty and deconditioning within this population, transcatheter interventions are growing in availability, yet remain very much in their infancy with a lack of long-term data (4).

## Case description

### Patient information

A 61-year-old male presented with a rectal mass during a primary care visit in 2021 and was then referred to the local surgical outpatient department (OPD). His past medical history was significant for varicose veins with previous radiofrequency ablation and avulsion. He was a retired bus driver and an ex-smoker with occasional alcohol use. A CT scan of the abdomen conducted in November 2021 showed a bulky rectal mass with liver and retroperitoneal involvement. The results of a liver biopsy showed a “well-differentiated neuroendocrine tumour,” and consequently, the local Medical Oncology service initiated treatment with lanreotide. During the subsequent 12 months of follow-up, he reported recurrent pedal oedema and dyspnoea, and was found to have a systolic murmur leading to a referral to the cardiology OPD for assessment. By this stage, everolimus was prescribed in addition to his lanreotide due to persistent symptoms of facial flushing and diarrhoea.

### Diagnostic assessment

**Figure 1:**
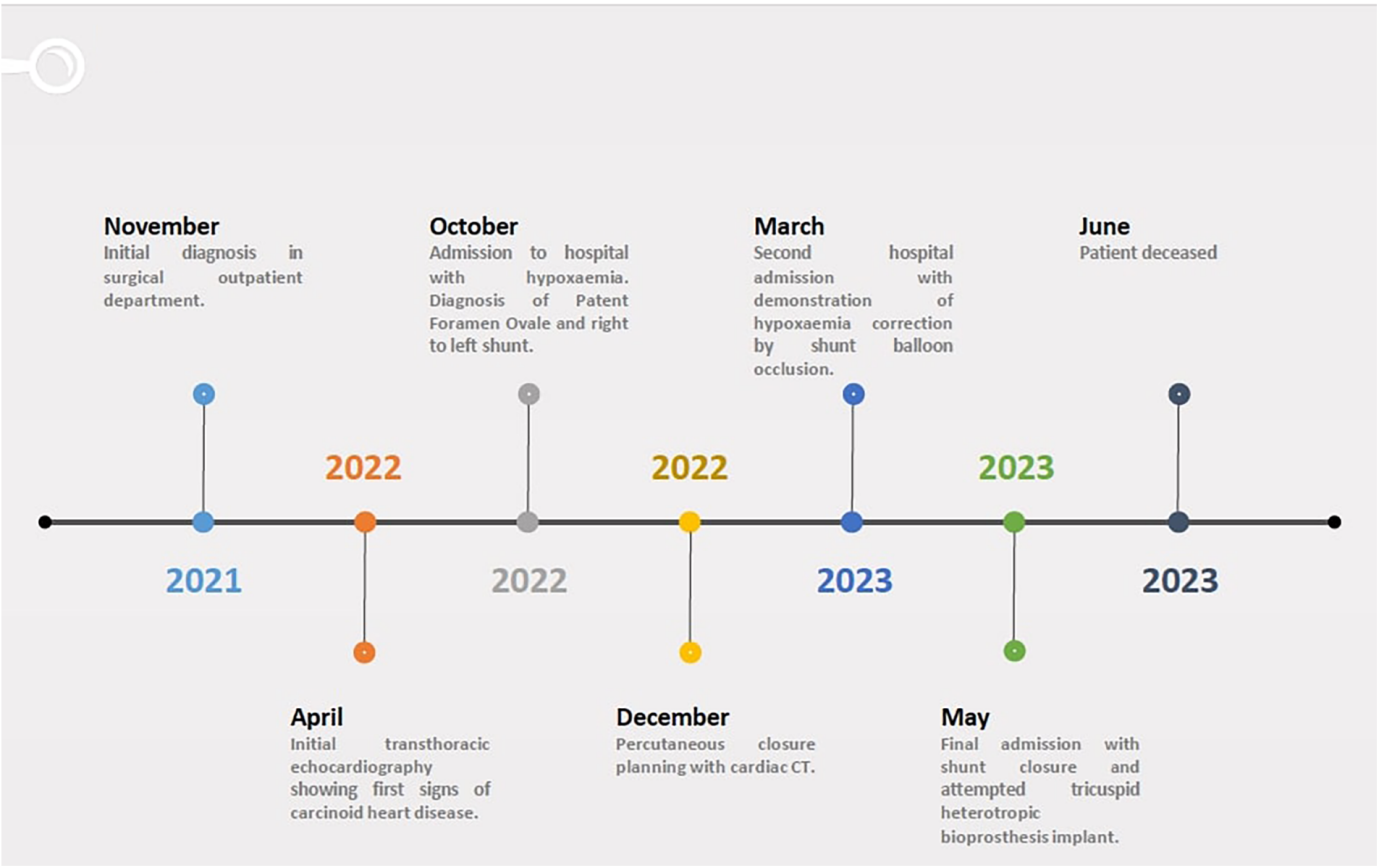
Timeline.

A full timeline of events can be seen in Figure 1. A TTE in April 2022 showed normal biventricular size and systolic function, with mild/moderate aortic incompetence (AI) and moderate/severe central TR. At this moment, the colour flow Doppler examination indicated that the intra-atrial septum (IAS) was found to be intact.

In early October of the same year, he needed to be admitted to the hospital due to an increasing need for oral diuretic. He had significant bilateral pedal oedema, bilateral basal lung crackles on auscultation, and an oxygen (O_2_) requirement of 12 L via nasal cannulae to maintain adequate saturations. He had obvious facial flushing and prominent neck veins.

His N-terminal pro-B type natriuretic peptide (NT-pro-BNP) was 582 pg/ml, haemoglobin 13.1 g/dl, creatinine 104 µmol/L, and cardiac troponin T 19 ng/L (<14). The lung parenchyma was clear on chest x-ray. The arterial blood gas (ABG) on 60% FiO_2_ showed mild respiratory alkalosis with type 1 respiratory failure (pH 7.5, paO_2_ 9.3kPa, pCO_2_ 4.9kPA, HCO_3_ 29.1 mEq/L, lactate 1.7 mmol/L). Electrocardiogram showed sinus tachycardia with right axis deviation. Viral panel was negative for seasonal variants. The CT pulmonary angiogram did not show any pulmonary embolus, vascular shunt, or interstitial lung disease to explain the hypoxia.

The updated TTE showed a preserved left ventricular function with interval RV dilatation exhibiting slightly reduced systolic function in the presence of severe TR, and a dilated right atrium (RA) (Supplementary Video S1). Upon evaluation by the clinical cardiology service, there was a suspicion of shunting across the IAS, and the TR trace by Doppler envelope was deemed insufficiently accurate for quantifying pulmonary arterial pressure. The presence of a right-to-left shunt was confirmed using a bubble study with agitated saline (Supplementary Video S2). The presence of cardiac platypnea-orthodeoxia was confirmed using supine ABG (paO_2_ 7.4 kPa, SpO_2_ 90.3%) compared with standing ABG (paO_2_ 6.9 kPa, SpO_2_ 87.5%).

To further clarify the suspicion of an intra-cardiac shunt, a TOE was performed (Supplementary Video S1). This showed a mobile IAS with PFO by a strongly positive bubble study both with agitated saline and agitated gelofusin. The mitral valve (MV) exhibited mild/moderate regurgitation (vena contracta, 0.2 cm), and the trileaflet aortic valve (AV) was determined to exhibit moderate AI secondary to a coaptation defect and a small focal prolapse of the non-coronary cusp. Both of these valves demonstrated leaflet thickening suggestive of carcinoid, primarily believed to be secondary to the significant right to left shunt. Both right-sided valves showed severe regurgitation, with a dilated tricuspid annulus with leaflet thickening and malcoaptation.

With the information gathered thus far, it was elected to perform left (LHC) and right catheterisation (RHC). LHC showed largely patent coronary arteries, with a dominant right coronary artery, left ventricular end-diastolic pressure (EDP) of 8 mmHg, and no aortic stenosis on catheter pull back. RHC was performed via the right median cubital vein. It demonstrated normal pulmonary arterial pressures (22/1/7 mmHg), normal pulmonary capillary wedge pressure (mean 3 mmHg), but elevated right filling pressures (RA pressure peak 14/mean 9 mmHg) and V wave consistent with a known TR. RV EDP was elevated at 10 mmHg, with pulmonary vascular resistance of 1 Wood unit. The Qp:Qs ratio was calculated at 0.93, and the cardiac output (CO) was mildly reduced (CO 3.53l /min, cardiac index 2.21l /min by thermodilution). There was no significant saturation step-up amongst the venous blood gases collected (PA 49.9%, IVC 56.3%, mid RA 48.2%, high RA 51.9%, SVC 48.6%), with a resting radial arterial saturation of 87% at rest and dropping to 84% during exercise.

At this stage, the patient was discharged with a prescription for long-term O_2_ therapy of 15 L O_2_ via nasal prongs and enrolment in the outpatient palliative care services. He was maintained on bumetanide 1 mg BD and eplerenone 25 mg as a diuretic regimen at this stage. The case was presented at a surgical conference, and secondary to frailty, he was considered unsuitable for surgery, albeit the medical oncology team determining that he had a reasonable prognosis for his carcinoid syndrome. Consideration was given to a percutaneous PFO closure with a tricuspid valve intervention concomitantly given the risk of worsening heart failure (the heart team perceived that the right chambers were offloading pressure via the PFO, and occluding this shunt may abruptly halt this compensatory mechanism, leading to circulatory collapse). To this end, a cardiac CT was undertaken for percutaneous procedural planning. This demonstrated mildly enlarged right heart chambers with annular dilatation, but without annular or tricuspid leaflet calcification.

On review in the OPD 5 months later, his dyspnoea and pedal oedema had continued to worsen. He had been reviewed at the structural cardiology OPD and listed for a balloon occlusion of the PFO to assess RA pressures and O_2_ saturations. It was felt that if the balloon occlusion improved the hypoxia, then an attempt at closure would be made if a heterotopic bioprosthesis was available.

### Therapeutic intervention

Late in March 2023, he was readmitted with worsening pedal oedema and hypoxia, with pre-syncope and hypokalaemia from diuretic therapy. He was transferred for the purpose of performing balloon occlusion on his PFO. He underwent a procedure where a 10 Fr sheath was inserted into his right common femoral vein to occlude his PFO for 3 min. During this period, his radial arterial oxygen saturation improved from 90% pre-procedurally (on room air) to 100%, with no change in his RA pressures before (26/14 mmHg) vs. after (25/12 mmHg) the procedure. Since the procedure was scheduled for diagnostic purposes rather than therapeutic purposes, a permanent closure device, sized by cardiac CT, was not made available on the same day. ECG-gated CT on that day demonstrated a stretched PFO of 10.4 mm. It was thought at this stage that his PFO was now confirmed to be the primary cause of hypoxia, in the context of severe TR with a low cardiac output and right to left shunting and normal PA pressures. Following optimisation of his fluid status, he was once again allowed to go home in order to provide time for procedural preparation.

After 2 months, he was readmitted secondary to carcinoid crisis, requiring coronary care unit (CCU) care with octreotide infusion. He remained markedly hypoxaemic despite receiving 100% FiO_2_ using high flow nasal cannulae. His medical condition was so severe that he needed the national inter-intensive care unit (ICU) transfer service to be transferred from his primary centre CCU to the structural intervention referral centre CCU for inpatient PFO closure and tricuspid valve heterotopic bioprosthesis insertion.

PFO closure was undertaken first, with bilateral femoral venous access, using intra-cardiac echocardiography for anatomo-morphological guidance. The PFO was crossed with a multipurpose catheter before the delivery of sheath for device deployment. Initial Amplatzer Talisman PFO 18 mm/18 mm Occluder (Model number 9-PFO-1818) was unable to oppose at the right atrial side, so a 30 mm/25 mm Amplatzer Talisman PFO Occluder (Model number 9-PFO-3025) was selected, with good opposition on both atrial sides (Supplementary Video S3). Occlusion was confirmed by venogram via pigtail in the hepatic veins near IVC/RA junction with no dye across the IAS (Supplementary Video S3). The O_2_ saturations immediately normalised, with a reduction in supplemental O_2_ requirements noted.

The procedure progressed to the deployment of the heterotopic bioprosthesis (“TricValve”). Attempts were made to advance the 27 Fr catheter for the deployment of the IVC valve against marked resistance. Pre-dilatation of the veins was attempted, without progression. The delivery sheath was removed and noted to be kinked and damaged at the tip. Due to operator concern, digital subtraction angiography was performed and confirmed extravasation of contrast medium from the external iliac vein (still can be observed in Supplementary Video S3). This was occluded with an 8 mm balloon; however, even at higher atmospheric pressures, this failed to contain the extravasation. Therefore, a 10 mm Bentley covered stent was deployed at 6 atm with good balloon expansion, and cessation of extravasation on venogram. Venography of the left iliac system demonstrated similar issues regarding vascular size and was deemed not large enough to receive the delivery sheath. The procedure was stopped at this junction to allow the stent to undergo maturation and endothelialisation, with an intention to resume the procedure in 4 weeks to pass the IVC and SVC delivery systems via femoral access.

### Follow-up and outcomes

Unfortunately, the patient became acutely delirious at the end of the case. A CT scan of the thorax/abdomen and pelvis showed a large haematoma in the right iliac fossa with multifocal infarction in the right kidney. A concomitant CT scan of the brain did not show acute pathology. In time, the patient was transferred back to his original hospital facility for ongoing CCU care. Ultimately, his condition proved fatal, with persistent delirium, intercurrent Clostridium difficile infection, and overwhelming carcinoid crisis leading to his premature demise. Upon discussion with the direct family members of the patient, autopsy was not undertaken.

## Discussion

Carcinoid tumours are a type of neuroendocrine tumours that are typically located in the bronchus and GI tract. Carcinoid syndrome is a clinical phenomenon secondary to the release of vasoactive substances [serotonin, 5-hydroxytryptamine (5-HT), 5-hydroxytryptophan, histamine, bradykinin, tachykinins, and prostaglandins], leading to vasomotor changes (secretory diarrhoea, bronchospasm, and hypotension) (5). The diagnosis of a carcinoid tumour involves 24 h collection of urine for 5-HIAA level (end product of serotonin metabolism), chromogranin A levels, PET-CT for tumour location, and tissue biopsy as appropriate. The treatment focuses on reducing proliferation of the primary tumour, and symptomatic control, predominantly via somatostatin analogues, e.g., lanreotide, octreotide (6).

Carcinoid heart disease (CHD) as a sequelae occurs eventually in approximately 20%–50% of patients with carcinoid syndrome and remains a major cause of morbidity and mortality. It is a recognised rare cause of right-sided valvular heart disease, particularly primary TR and stenosis, with development of plaque-like, fibrous endocardial thickening on valves (mostly tricuspid and pulmonary due to metabolism of serotonin in the lung parenchyma). Clinical features include dyspnoea, fatigue, systolic murmur best heard along the sternal border, elevated jugular venous pressure (JVP) with a prominent V wave, ascites, and peripheral oedema (7). The involvement of the left-sided heart valves is uncommon due to the metabolism of 5-HT within the lungs, occurring in approximately one-third of patients. The reasons for left-sided valve disease include a PFO, bronchial carcinoid, or very high levels of 5-HT.

The tricuspid valve itself is the largest and most apically placed heart valve. There is a low-pressure difference between the right atria and ventricle with gradients across the valve expected to be <2 mmHg. It is thought of as four components: leaflets, papillary muscles, chordal attachments, and annulus, with typically three leaflets (anterior, posterior, and septal), but may be bicuspid or have more than three leaflets. Its complex anatomy is essential to understanding the pathophysiology of TR in carcinoid heart disease. Annular dilatation, right atrial and ventricular dilatation, and septal bowing are all recognised features within the clinical syndrome (1).

Symptomatic primary severe TR should have a surgical replacement as per the 2021 ESC/EACTS VHD guidelines (8). However, there is limited specific advice regarding CHD management in these guidelines. Overall, there is limited data, but the existing data indicate a poor outlook. The largest series published in 2019 (2) showed that over 30 years, 240 patients with CHD who underwent valvular heart surgery at Mayo Clinic had high peri-operative morbidity and mortality, particularly when RV dysfunction is present either pre- or post-operatively. The authors quoted a 2-year overall survival of 60%, and a 5-year survival of 34%, albeit with a reduction in early post-operative mortality in more recent times. Low operative numbers (TVR and PVR are relatively uncommon in adult cardiothoracic surgical practice) result in limited information regarding surgical technical aspects and valve replacement optimisation (3). Lastly, it is recognised that the implantation of bioprosthetic material in the context of active carcinoid is commonly associated with valve degeneration, particularly pulmonic implants (9).

PFO closure in isolation has been shown to benefit patients with carcinoid syndrome without valvular disease at 6 months regarding systemic arterial oxygenation, 6 m walk test, and New York Heart Association score (10). However, prospective data regarding the management of PFO and valvular heart disease are restricted to isolated case reports. This does not negate the value in the correction of carcinoid heart disease sequelae, as we have demonstrated an improvement in the symptoms and hypoxaemia of the patient in this case, as was also shown in other similar clinical scenarios (10). After correcting the hypoxaemia in this patient population, staged valvular repair/replacement can be considered at a subsequent junction.

We would suggest a multi-modal imaging approach to the evaluation of these patients, as demonstrated by the initial diagnosis and evaluation of the aetiology of valvular disease via transthoracic and transoesophageal echocardiography. Fluoroscopy, along with right heart catheterisation, is essential for shunt evaluation, and angiography is necessary for the exclusion of concomitant coronary artery disease. These procedures remain an integral part of the diagnostic pathway. ECG-gated cardiac CT is complementary for procedural planning for sizing of PFO closure device, and for the evaluation of tricuspid valve leaflet calcification if subsequent transcatheter correction is anticipated. Finally, we would suggest an expedited approach as feasible for each medical facility in the diagnostic work-up and therapeutic intervention for this patient population, as demonstrated by the rapid deterioration of our patient throughout their clinical course.

### Take home points

Carcinoid heart disease is a rare cause of right-sided valvular heart disease, particularly tricuspid regurgitation and stenosis. We demonstrate a case of carcinoid heart disease complicated by TR causing right to left heart shunting via a stretched PFO, with marked hypoxia and severe heart failure. We wish to highlight the role that multi-modality imaging played during this patient’s journey. Unfortunately, there are limited surgical data regarding CHD, with poor outcomes in advanced disease and higher peri-operative complications. We encourage considering percutaneous palliative procedures as, albeit for a short period of time, the patient in this case improved subsequent to such interventions.

## Data Availability

The original contributions presented in the study are included in the article, further inquiries can be directed to the corresponding author/s.
